# Characterization and Analysis of Okoume and Aiele Essential Oils from Gabon by GC-MS, Electronic Nose, and Their Antibacterial Activity Assessment

**DOI:** 10.3390/s20236750

**Published:** 2020-11-26

**Authors:** Youssra Aghoutane, Mohammed Moufid, Soukaina Motia, Guy Stephane Padzys, Linda Priscilia Omouendze, Eduard Llobet, Benachir Bouchikhi, Nezha El Bari

**Affiliations:** 1 Biosensors and Nanotechnology Group, Department of Biology, Faculty of Sciences, Moulay Ismaïl University, B.P., Zitoune, Meknes 11201, Morocco; youssi.aghoutane@gmail.com (Y.A.); mohammed.moufid.2@gmail.com (M.M.); sokainasafae@yahoo.fr (S.M.); 2 Sensor Electronic & Instrumentation Group, Department of Physics, Faculty of Sciences, Moulay Ismaïl University, B.P., Zitoune, Meknes 11201, Morocco; benachir.bouchikhi@gmail.com; 3 Department of Biology, Faculty of Sciences, University of Sciences and Technolgy of Masuku, Franceville 901, Gabon; padzys@gmail.com (G.S.P.); Priscilia.Omouendze@gmail.com (L.P.O.); 4 Department of Electronic Engineering, Universitat Rovira i Virgili, MINOS-EMaS, Microsystems and Nanotechnologies for Chemical Analysis, Avinguda Països Catalans, 26, 43007 Tarragona, Spain; eduard.llobet@urv.cat

**Keywords:** okoume, aiele, essential oils, electronic nose, gas chromatography-mass spectrometry, antimicrobial activity

## Abstract

Essential oil resins of *Aucoumea klaineana* (Okoume) and *Canarium schweinfurthii* (Aiele) species, of the Burseraceae family, were studied to investigate their bioactive constituents and their antibacterial activities. Aiele resin had a higher yield (6.86%) of essential oil than Okoume (3.62%). Twenty-one compounds for Okoume and eighteen for Aiele essential oil were identified using a gas chromatography-mass spectrometry (Gp-C-MS) technique. The main compounds identified in Okoume essential oil were benzenemethanol, α, α,4-trimethyl (28.85%), (+)-3-carene (3,7,7-trimethyl bicyclo[4.1.0]hept-3-ene) (17.93%), D-Limonene ((4R)-1-methyl-4-prop-1-en-2-ylcyclohexene) (19.36%). With regard to the Aiele essential oil, we identified (1R,4S)-1-methyl-4-propan-2-ylcyclohex-2-en-1-ol (26.64%), and 1-methyl-4-propan-2-ylcyclohex-2-en-1-ol (26.83%). Two strains of bacteria, *Escherichia coli* and *Staphylococcus aureus*, were used in antibacterial tests. *S. aureus* was found to be more sensitive to Okoume and Aiele essential oils, with a high inhibition zone ranging from 20 to 16 mm. In comparison, the inhibition zone ranged from 6 to 12 mm for *E. coli*. An electronic nose (e-nose) combined with pattern analysis methods such as principal component analysis (PCA), discriminant function analysis (DFA), and hierarchical cluster analysis (HCA) were used to discriminate the essential oil samples. In summary, the e-nose and GC-MS allowed the identification of bioactive compounds in the essential oil samples, which have a strong antimicrobial activity, with satisfactory results.

## 1. Introduction

The Burseraceae are a medium-sized family comprising about 700 species in 17 to 19 genera. The family includes both trees and shrubs [1]. It is endemic to the tropics of Africa, Asia, America, and subtropics. It is also represented by a few taxa in some warm temperate regions. In this family, resin plants produce essential oils, which are mainly extracted from the leaves, bark, and oleaginous resins. These essential oils, also called volatile or ethereal oils [2], are aromatic oily liquids rich in many bioactive compounds, which have an important biological role as antibacterial [3], antifungal [4] agents, and are responsible for many medicinal properties [5]. *Aucoumea klaineana* Pierre, or okoume as it is commonly known in Gabon, and the species *Canarium schweinfurthii* or aiele, are the two most important wood species in Gabon [6].

The natural range of okoume is limited to western and central Gabon and the regions of Equatorial Guinea, the Republic of Congo, and Cameroon [7]. The *Canarium schweinfurthii* or aiele tree grows in the equatorial forest region of Cameroon, Central African Republic, Gabon, and Congo [8]. All these species produce a gum that solidifies into resin [9]. The resin is distilled to produce essential oil commonly used in traditional medicine and perfumery because of its lavender smell. Okoume resin is used to purify water, to ripen abscesses, and is applied as a substitute for incense. The bark can be employed as an antiseptic, astringent, or antidiarrheal agent [10,11]. In addition, aiele resin is used in traditional medicine for the treatment of various diseases such as cutaneous lesions and microbial infestations. It is also used for its emollient, stimulating, and diuretic properties [12].

Several methods have been proposed to identify volatile compounds contained in essential oils such as high-precision liquid chromatography (HPLC) [13], gas chromatography-mass spectrometry (GC-MS) [14], GC–MS combined with chemometric resolution methods [15], Two-Dimensional Gas chromatography (GC × GC) [16], and ATR-Fourier transform mid-infrared spectroscopy (FT-IR) [17]. However, most of these techniques are usually time-consuming and costly. Electronic noses have been explored as a more affordable approach to discriminate different essential oils [18].

The electronic nose (e-nose) is a device that integrates a group of gas sensors with partial specificity and an adequate pattern recognition system, able to recognize both simple and complex odors [19]. In e-nose devices, samples are analyzed and classified (in terms of quality) on the basis of their fingerprints or patterns obtained from their volatile compounds [20]. Electronic noses are fast, economical, convenient, and reliable devices for non-destructive testing [21]. Electronic noses are used for many applications such as product classification within the food industry sector [22,23,24], determination of the quality and storage life [25,26], recognizing the maturity of fruit [27,28,29], and assessment of adulterated products [30,31,32,33]. Many studies on the use of e-nose systems for assessing the quality and distinction of natural MAPs (medicinal and aromatic plants) have been described in the literature [34]. For instance, the quality control of *Alpinia officinarum* using an e-nose and GC-MS coupled with chemometrics [35], the discrimination and characterization of licorice (*Glycyrrhiza glabra* L.) roots utilizing electronic nose and HS-SPME/GC/MS analysis [36], as well as the quality control method for musk by an e-nose coupled with chemometrics [37]. Such an approach has also been envisaged for some other plants, including *Mentha spicata* L. [38], *Cymbidium ensifolium* [39], ginseng [40,41,42], *Eurycoma longifolia* [43], *apiaceae* [44], *Cannabis sativa* L. [45], *Glycyrrhiza glabra* L. [46], *Citrus reticulata* [47], as well as the classification of rosemary essential oil (*Rosmarinus officinalis* L.) [48].

The essential oils of resin contain a high percentage of compounds with known antibacterial and antifungal properties. Nowadays, bacterial infections remain a major cause of morbidity and mortality in humans and animals. The phenomenon of antibiotic-resistant bacteria continues to increase in frequency and number worldwide, and new resistance problems have recently emerged, which further complicates and hinders the treatment of critical infectious diseases [49]. Besides, the use of natural products such as essential oils as antimicrobial compounds has many advantages. These comprise fewer adverse effects, better patient tolerance, relative inexpensiveness, and wide acceptance because of their traditional applications, renewable nature, and better biodegradability [50].

To the best of our knowledge, no attempt has yet been made to investigate the compounds of okoume and aiele essential oils using (e-nose) combined with GC-MS. For these reasons, on the one hand, we investigated the capability of an e-nose coupled to various chemometric methods, namely principal component analysis (PCA), discriminant function analysis (DFA), and hierarchical cluster analysis (HCA), to first classify and discriminate between the two Gabonese essential oils of *Canarium schweinfurthii* (aiele), and *Aucoumea klaineana* (okoume). In addition, we determined the chemical composition and evaluated the efficacy and pure major compounds by GC-MS analysis. On the other hand, we used the aromatogram method to reveal their antibacterial activity in vitro against two bacteria strains *Escherichia coli* (Gram−) and *Staphylococcus aureus* (Gram+).

## 2. Materials and Methods

### 2.1. Sample Preparation

In this paper, two types of Gabonese essential oils called okoume and aiele of the medicinal plant family Burseraceae, were used. *Aucoumea klaineana* (okoume) and *Canarium schweinfurthii* (aiele) resins were collected in March 2017 in Djoutou South East Gabon. The resins were hydro-distilled for 3 h in a Clevenger-type apparatus. The essential oils were dried after decantation using anhydrous sodium sulfate, then filtered and stored under dark conditions at 4 °C before analysis. The percentage yield of essential oils has been calculated on the basis of the ratio (mass of dry oil extracted to mass of resin).

For performing the measurements, 0.5 mL of each essential oil were heated to 32 ± 1 °C, inside a thermostatically controlled sampling chamber for a headspace generation time of 10 min in order to analyze them using the e-nose system. These samples were subject to heating in a thermostatic bath at 95 °C for 20 min for performing the GC-MS analysis.

### 2.2. GC-MS Measurements

The chemical analysis of the volatile atmospheres resulting from the essential oils was carried out using GC-MS. The analyses employed two instruments, a GC series 6890 with liquid automatic sampler series HP7683 coupled to a mass spectrometer 5972 (Agilent Technologies Inc., Santa Clara, CA, USA). The column used in chromatography was an HP5 analytical column (Agilent Technologies Inc.), with a length of 30 m, an internal diameter of 0.25 mm and a 0.25 μm thick stationary phase. In this case, the injections of the samples were done manually. The samples were weighed in vials of 10 mL. Subsequently, a manual injection syringe was introduced into the headspace and the volatile sample was injected into the GC. The analysis method was as follows: injector: splitless; inlet pressure: 11.03 psi; inlet temp. 220 °C; column flow 1.3 mL/min; mode: constant flow; volume: 500 μL; temperature: initial T: 60 °C, 2 min; ramp 3 °C/min up to 90 °C, 9 min; ramp 20 °C/min up to 220 °C, 2 min; Analysis time: 20.5 min; acquisition in full scan mode and range from 15 to 300 *m*/*z*. The mass spectra obtained from our GC-MS system were compared to those of the NIST11 MS database. Volatile compounds were tentatively identified via comparison of the spectrum recorded and those in the database when a high degree of similarity was found (equal to or higher than 80%).

### 2.3. Antibacterial Assay

#### 2.3.1. Preparation of Bacterial Inoculum

The antibacterial activity was performed on two strains of Gram-negative (*E. coli* (ATCC 25922)) and Gram-positive (*S. aureus*) bacteria, obtained from the microbiology laboratory of the Faculty of Science, Moulay Ismail University of Meknes (Morocco). The bacteria to be tested were sown on Petri dishes containing selective media adapted to the bacterial strains used and incubated at 37 °C for 24 h to obtain well-isolated colonies. After this incubation time, 1 to 3 well isolated and perfectly identical bacterial colonies were collected using a platinum loop, then emulsified in a tube containing 2 mL of 0.9% sterile physiological water and vortexed. The inoculum was adjusted to 0.5 McFarland, corresponding to an optical density of (0.08 to 0.10) at 625 nm [51].

The inoculum thus obtained was diluted in sterile physiological water depending on the type of germs:

- For Enterobacteriaceae, 100 µL of inoculum were diluted in 10 mL of physiological water.
- For Staphylococcus, 1000 µL of inoculum were diluted in 10 mL of physiological water.

This dilution allowed us to have a bacterial suspension at 107 CFU/mL.

#### 2.3.2. Antibacterial Testing

The antibacterial activity of different essential oils was evaluated by the aromatogram method [52]. Briefly, the test was performed in sterile Petri dishes (100 mm diameter), previously poured by media (Müller Hinton ((Sigma Aldrich, Saint Louis, MO, USA) for bacteria), seeded with a microbial suspension (107 cell/mL). Then, sterile 6 mm diameter Wattmen paper discs, soaked with 10 µL of each essential oil, were placed sterile on the surface of the culture media. After incubation for 24 h at 37 °C, the results are read by measuring the diameter, in mm, of the inhibition zone. All experiments were repeated three times [51].

### 2.4. Electronic Nose Setup Measurements

An electronic nose system based on six semiconductor gas sensors obtained from Figaro Engineering, Inc. (Osaka, Japan). (Table 1) were used to analyze the two essential oils, okoume and aiele.

**Figure 1:**
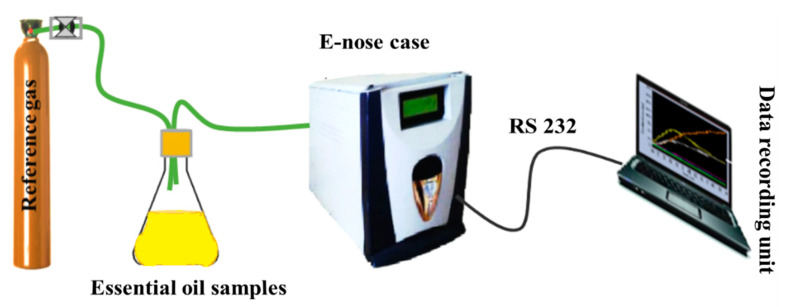
E-nose setup for essential oils (okoume and aiele) analysis.

The experimental system is mainly composed of three parts: a sampling unit, the sensor array, and a data acquisition system. An overall view of the system is shown in Figure 1. The data acquisition system registers the electrical signals generated from gas sensors due to the interaction of their sensitive surfaces with the volatile organic compounds (VOCs) released from essential oils. Each sample was placed in a 100 mL airtight glass vial with two small holes in their cover to allow the headspace to be analyzed with the e-nose equipment. Six replicates of each essential oil headspace (okoume, aiele) sample were measured without any pre-treatment.

### 2.5. Statistical Analysis

#### 2.5.1. Feature Extraction

Feature extraction is an essential pre-processing step for pattern recognition [31,53]. The functions used for data analysis are automatically extracted from the gas sensor responses in order to extract the maximum amount of information available in the database. Consequently, for each measurement performed, four representative characteristics were extracted from the response of each sensor, which are as follows:

- G0, the initial sensor conductance determined as the average value of its conductance during the first minute of a measurement.
- Gs, which represents the steady state conductance determined as the average value of its conductance during the last minute of a measurement.
- dG/dt, which corresponds to the dynamic slope of the conductance determined between 2 and 9 min of measurement.
- A, the area under the conductance curve during a defined time interval between 2 and 9 min of measurement. This area has been estimated by the trapezium method.

#### 2.5.2. Data Processing

To evaluate the performance of the e-nose for the discrimination between the two Gabonese essential oils, multivariate unsupervised and supervised data analysis methods such as PCA, DFA, and HCA were applied.

Radar plots are particularly appropriate to illustrate the overall responses of the sensor system. A Radar plot is used to display the results generated by several sources and is strongly recommended when associated with a statistical study, since it frequently anticipates the classification of data groups. Unit radius radar plots were used in this study to check for differences between the two samples of okoume and aiele essential oils. This makes it easy to accurately visualize the differences between typical plots.

PCA is a powerful, unsupervised linear pattern recognition technique that has proven effective in easily visualizing the maximum amount of information in a data set. It decomposes the primary data matrix by projecting multidimensional data into a new coordinate database formed by orthogonal directions with maximum data variation.

The DFA method is the most frequently used and best-studied supervised pattern recognition method [54]. It is based on the determination of discriminant functions, which maximize the variance ratio between classes by minimizing the intra-class variance ratio. This technique constitutes the factorial method. Its objective is to examine whether or not the variables are sufficient to be able to classify correctly the data a posteriori into their a priori groups.

Finally, HCA refers to the unsupervised classification of patterns (vectors of characteristics) into groups (clusters) such that individuals in the same group are more similar than those in different groups [31]. In HCA, individuals are often represented in a dendogram. The distance between two objects in a dendrogram is evaluated to determine the similarity of the objects according to each of their attributes.

## 3. Results and Discussion

### 3.1. Essential Oil Yield of Two Species

The yields of essential oils for the two species selected in this work were 3.62% for *Aucoumea klaineana* resin and 6.86% for *Canarium schweinfurthii* resin. The essential oil yield of *Canarium schweinfurthii* resin was significantly higher than that of the *Aucoumea klaineana.*

### 3.2. GC-MS Results

The qualitative analysis of VOCs from the headspace of essential oils (okoume, aiele) was performed using GC-MS to identify the main compounds responsible for their benefits on well-being and health.

**Figure 2:**
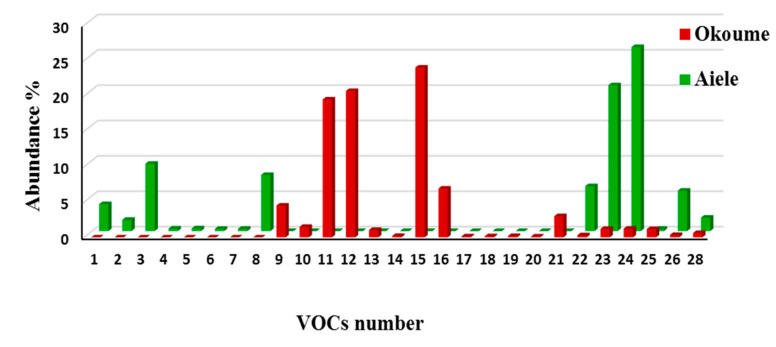
Histogram of the identified volatile compound of aiele and okoume essential oils by GC-MS analysis. The VOC numbers correspond to those used in Table 2.

**Figure 3:**
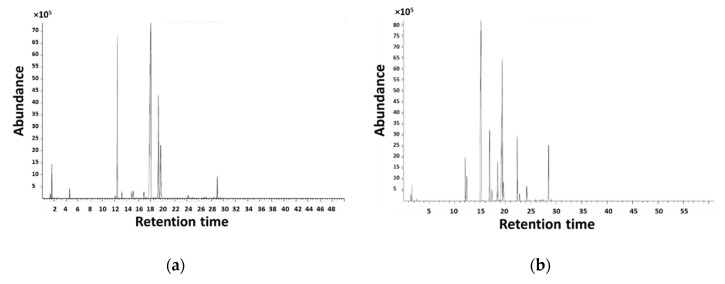
Total ion current (TIC) chromatogram of: (**a**) okoume and (**b**) aiele essential oils.

Figure 2 and Figure 3 show that there are VOCs that were specific to an essential oil, and other VOCs that appear in the headspace of both essential oils. The amount of VOCs released by okoume essential oil samples was much higher than that of the aiele essential oil samples. Mass spectra were compared to the NIST11 mass spectra database and a compound was considered as identified provided that the match between our experiment and the database was 75% or higher. Using this technique, twenty-one compounds were identified from okoume essential oil, compared to eighteen compounds from aiele (Figure 2).

Table 2 lists the main compounds that are tentatively identified in each sample, indicating their CAS number and chromatographic retention time (depending on the analytical method used). The degree of similarity of their spectra is also indicated.

Figure 3 shows that the chromatogram of okoume essential oil showed a significantly higher number of peaks than that of aiele essential oils. The first compound appeared at 1.63 min. Then a succession of peaks of notable height appeared placed between different very small peaks. In all, there were four important peaks, between t = 10 min and 20 min. In addition to this, there are five dominant peaks that appeared at about 15 min and 28.5 min. Finally, other peaks with very small amplitudes were obtained. According to these results, the main components were α-Terpineol (2-(4-methylcyclohex-3-en-1-yl)propan-2-ol) (29.04% and 28.49% for okoume and aiele essential oils, respectively), which was recorded in other studies on the composition of the Burseraceae family [55]. Benzenemethanol, α, α, 4-trimethyl (28.85%), (+)-3-Carene (3,7,7-trimethyl bicyclo[4.1.0]hept-3-ene) (17.93%), D-Limonene ((4R)-1-methyl-4-prop-1-en-2-ylcyclohexene) (19.36%) for okoume essential oil. This result has been confirmed by previously published research conducted on the volatile oil of *Aucoumea klaineana* oleoresin harvested in Gabon [56]. (1S,4R)-4-Isopropyl-1-methyl-2-cyclohexen-1-ol) (25.90%) and 2-cyclohexen-1-ol, 1-methyl-4-(1-methylethyl)-, cis(1-methyl-4-propan-2-ylcyclohex-2-en-1-ol) (26.83%) were identified for aiele essential oil. These results are also in good agreement with previous works on the composition of essential oils in *Canarium schweinfurthii* (aiele) resin from Gabon [57]. Other predominant constituents were benzenemethanol, α, α, 4-trimethyl-(28.85%), 1, 3, 8-p-Menthatriene(1-methyl-4-prop-1-en-2-yl-cyclohexa-1,3-diene) (24.91%), bicyclo[2.2.1]heptan-2-one, 1,7,7-trimethyl-, (1S)-Camphor (26.92%) for okoume and 1-methyl-4-propan-2-yl-cyclohexa-1,4-diene (22.31%), 2-methyl-5-propan-2-yl bicyclo[3.1.0]hexan-2-ol (24.7%), (4-methyl-1-propan-2-yl cyclohex-3-en-1-yl) acetate (26.64%) for aiele. As can be derived from Table 2, the predominant shared compounds in aiele and okoume essential oils are o-cymene(1-methyl-2-propan-2-ylbenzene), (+)-4-Carene ((1R,4S,6S)-4,7,7-trimethylbicyclo[4.1.0]hept-2-ene).

### 3.3. Antibacterial Activity

**Figure 4:**
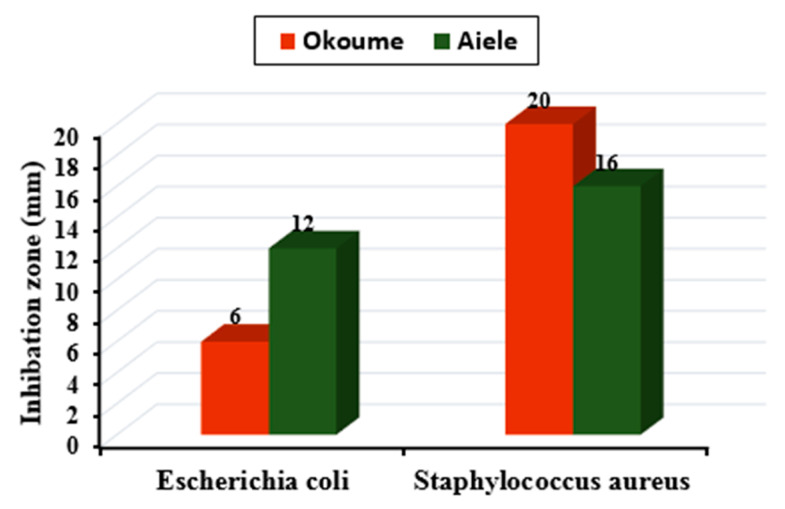
Antimicrobial effects of okoume and aiele essential oils on two pathogenic bacteria.

The results of the antibacterial test are summarized in Figure 4. They show that both the essential oil of okoume and aiele have a broad antibacterial activity because they inhibit the growth of Gram+ and Gram− bacteria, producing a zone of inhibition diameter (zdi) of 6 to 12 mm for *E. coli* and 16 to 20 mm for *S. aureus*, respectively. According to the results in Figure 4, *S. aureus* is sensitive to both essential oils. However, the inhibition zone is larger for the oil extracted from *Aucoumea klaineana* (okoume) than for the oil extracted from *Canarium schweinfurthii* (aiele). Based on these results, it can be stated that the essential oils showed a clear antimicrobial activity on all strains tested.

Okoume essential oil has a significant inhibitory effect against *S. aureus* (20 mm) and a moderate inhibitory effect for *E. coli* (6 mm). In the literature, essential oils with higher concentrations of components including: δ-3-carene, linalool, α-pinene, β-pinene, limonene, 1, 8-cineole [58], and terpineol [59] have been reported to show enhanced antibacterial activity. These findings are consistent with the results reported here for the essential oil extracted from *Aucoumea klaineana* (okoume).

An inhibitory effect for both strains was also observed for aiele essential oil. The literature shows that the presence of oxygenated monoterpenes, including 1, 8-cineole, linalool, -terpineol, nerolidol, and spathulenol, in high proportions results in antibacterial properties [60]. The essential oil of aiele is composed of relatively low proportions of some of these compounds and has been reported to show inhibitory activity. These reports are consistent with the results presented here. In particular, essential oils consist of complex mixtures of many constituents. The possible synergistic effects of the constituents present in the essential oil must also be taken into account. As mentioned above, okoume and aiele essential oils have a strong antimicrobial activity against these two bacteria: *S. aureus* (Gram+) and *E. coli* (Gram−). According to GC-MS results, okoume essential oil contains a high percentage of d-Limonene ((4R)-1-methyl-4-prop-1-en-2-yl-cyclohexene) (19.36%), which is absent in aiele essential oil. This compound is considered to be the most toxic against *S. aureus* in comparison to other terpenes present [61]. A similar finding was reported for d-limonene-containing nano-emulsion, where 12.5 μg mL^−1^ was required to inhibit *E. coli*, whereas Gram+ *S. aureus* bacteria required only 3.125 μg mL^−1^ [62]. Our results confirm a higher antibacterial effect of okoume on *S. aureus* compared to Gram− bacteria (*E. coli*). This can also explain the strong sensitivity of *S. aureus* (Gram+) to Okoume and not to Aiele essential oil. Generally, the main antimicrobial active components, act against the cytoplasmic membrane of bacteria cell. The main mechanisms of action of okoume essential oil compounds (d-limonene (((4R)-1-methyl-4-prop-1-en-2-yl-cyclohexene), p-cymene (1-methyl-4-propan-2-ylbenzene) and 3-carene (3,7,7-trimethyl bicyclo[4.1.0]hept-3-ene)) are as follows. D-limonene ((4R)-1-methyl-4-prop-1-en-2-ylcyclohexene) acts against the cytoplasmic membranes of microorganisms, resulting in loss of membrane integrity, thus modifying its permeability and leading to the leakage of certain cellular components such as ions and proteins, dissipation of proton motive force and inhibition of respiratory enzymes [63]. Cymene can also cause an expansion of the cytoplasmic membrane, inducing a reduction of the membrane potential. Another study [64] suggested that the antimicrobial effects of p-cymene (1-methyl-4-propan-2-ylbenzene) may be due to a disruption of lipids in the bacterial membrane. p-cymene (1-methyl-4-propan-2-ylbenzene) may also have an impact on protein synthesis and cell motility, which was recently studied in *E. coli* O157:H7 [65]. Morphological changes induced by p-cymene (1-methyl-4-propan-2-ylbenzene) treatment have also been studied, showing damage in the morphological structure of the cell [66]. Similarly, 3-carene (3,7,7-trimethyl bicyclo[4.1.0]hept-3-ene) can damage the cell wall and cell membrane of Gram+ and Gram− bacteria, leading to leakage of protoplasmic contents [67] and reduction of proteins [68]. This, in turn, kills the bacteria and causes rapid degradation of ATP. Previous studies indicate that the sharp decrease in bacterial ATP concentration with the addition of 3-carene (3,7,7-trimethyl bicyclo[4.1.0]hept-3-ene) could be due to the inhibition of ATP synthesis, increased ATP hydrolysis, and interruption of biofilm structure, confirming damage to bacterial energy metabolism and biosynthetic pathways [69].

The antimicrobial activity of both essential oils (Okoume and Aiele) against the (Gram−) bacteria could be associated to the presence of monoterpenes (α-terpinene (1-methyl-4-propan-2-ylcyclohexa-1,3-diene)), 4-carene ((1R,4S,6S)-4,7,7-trimethylbicyclo[4.1.0]hept-2-ene), *p*-cymene (1-methyl-4-propan-2-ylbenzene), (+)-3-Carene (3,7,7-trimethyl bicyclo[4.1.0]hept-3-ene). Previous studies indicated that these compounds have synergistic antibacterial activity. Another study [70] examined the essential oil of *Glossogyne tenuifolia*, and its various compounds (including *p*-cymene (1-methyl-4-propan-2-ylbenzene)) against some common food pathogens. *p*-cymene (1-methyl-4-propan-2-ylbenzene) was found to be the most common compound in this oil. A minimum bactericidal concentration of 12 mg/mL of *p*-cymene (1-methyl-4-propan-2-ylbenzene) completely inhibited *E. coli* O157:H7, and *S. aureus* showed a minimum microbicidal concentration of 6 mg/mL, in addition *p*-cymene (1-methyl-4-propan-2-ylbenzene) can induce significant synergistic antibacterial activity with other compounds such as limonene and α-terpinene. That increased the antibacterial effect against pathogenic bacteria [71]. In addition, the low sensitivity of Gram− bacteria compared to the Gram+ bacteria found for both essential oils is probably due to the presence of an external lipopolysaccharide membrane that acts as a physical barrier to lipophilic compounds, including essential oils [72]. Further studies on the combination of these essential oils with conventional antibiotics are needed to offer a promising and significant potential for the development of new therapies in the treatment of infectious diseases caused by multi-resistant microorganisms.

### 3.4. Electronic Nose Results

#### 3.4.1. Sensor Responses

**Figure 5:**
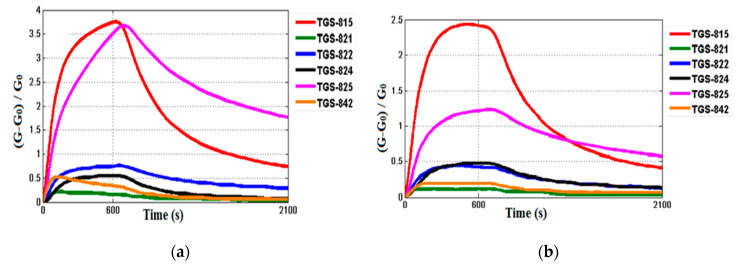
Sensor electrical conductance changes in the presence of (**a**) okoume, (**b**) aiele essential oils using an electronic nose system.

The volatile compounds generated from the essential oil were pumped with a flow rate of 100 mL/min through the measurement chamber containing the sensor array. Upon injecting the sample, data were acquired every 2 s over 10 min. Figure 5 shows typical signals of the normalized conductance of the six TGS gas sensors exposed to the headspace of the two types of essential oils from the same family plants, okoume (Figure 5a) and aiele (Figure 5b). Among the six different gas sensors present in the array, the TGS 815 gas sensor showed the highest response to the two types of essential oils.

#### 3.4.2. Radar Plot Representation

**Figure 6:**
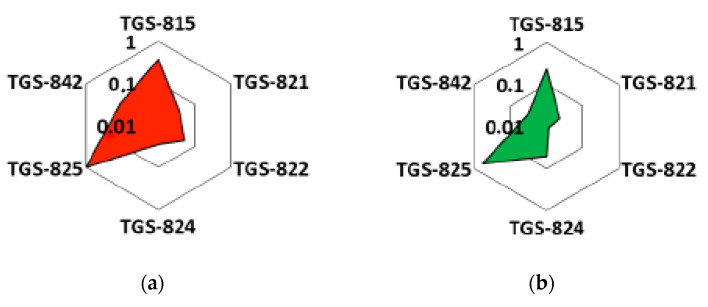
Radar plots of (**a**) okoume, (**b**) aiele essential oils using the conductance change (Delta-G) of sensors as response feature.

Radar-plots with unitary radius were used to observe whether pattern differences (i.e., fingerprints) developed between essential oil samples. As can be seen, the radar plots (Figure 6) show a clear variation between two essential oils (okoume, aiele). To construct these plots, the values of delta G for the sensor responses were divided by the value corresponding to sensor TGS 825, which showed the maximum signal. This helped to visualize the differences among typical response patterns. Indeed, a clear pattern variation (fingerprint) exists between okoume and aiele, which confirms the ability of the e-nose to easily discriminate between the two types of essential oils using the radar plot representation.

#### 3.4.3. Principal Component Analysis

**Figure 7:**
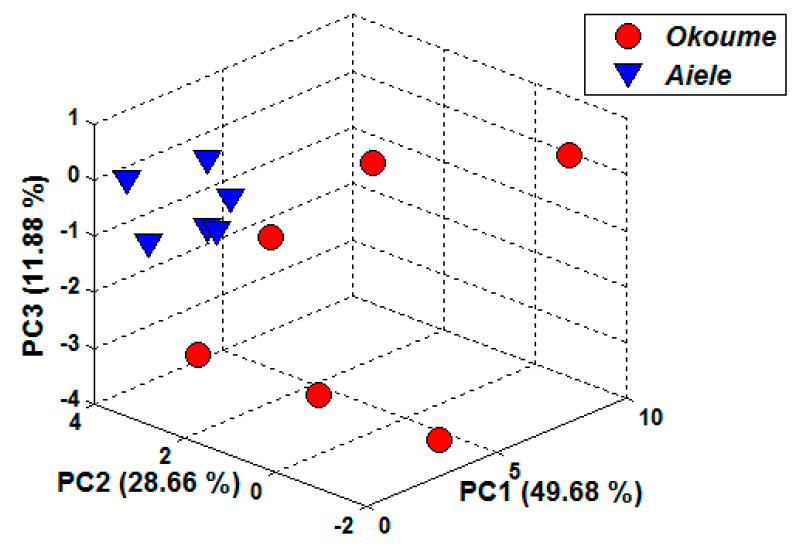
Three-dimensional PCA plot performed on essential oils, namely, okoume and aiele, using the conductance change (Delta-G) of sensors as a response feature.

The PCA method was applied as an exploratory technique to study the clustering of data points in the multidimensional dataset. Before approaching the different classification tasks by PCA, it is necessary to reduce the size of the data matrix by selecting variables containing differential information, as the number of variables selected depends on the classification task [60]. In a first step, the data matrix was analyzed using the PCA technique to characterize and discriminate okoume and aiele essential oils. Figure 7 shows the projections of the experimental results on a three-dimensional (3D) graph using the first three new main components. Showing a cumulated variance of the first three principal components explain 49.56%, 28.66%, and 11.88%, respectively (i.e., 90.22%) of the total data variance. From this figure, it can be noticed that the two types of essential oils can be perfectly discriminated by the e-nose in spite of the method uncertainty (i.e., variability within each class) caused mainly by variability in sensor response. On the one hand, the loadings matrix of (PC) was carried out using three features (normalized delta G, slope, and area) multiplied by the six sensors. The score plot is shown in Figure 7. Moreover, the discrimination obtained by the PCA is in good concordance with the specific VOCs that are identified by GC-MS in each essential oil. In particular, okoume essential oils contained some distinctive volatiles (i.e., acetone, toluene, pinenes, and terpenes) that do not appear in the essential oil of aiele. Sensor TGS 822 is quite sensitive to organic solvents such as toluene and acetone and, indeed the response of this sensor to volatiles from okume is significantly higher than for volatiles from aiele (see Figure 6). These differences may explain the discrimination between the two essential oils reached by the e-nose instrument using PCA.

**Figure 8:**
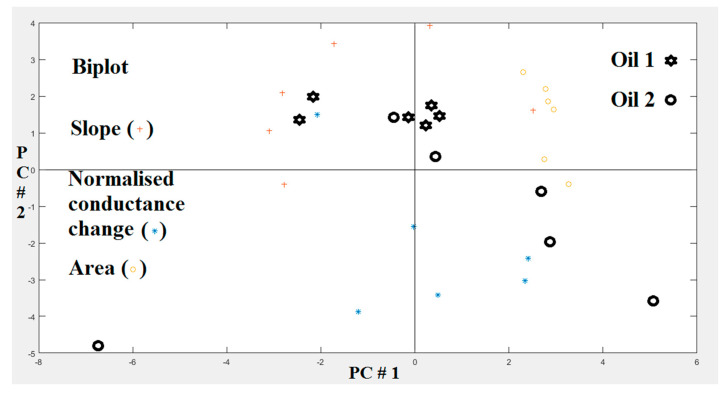
PCA biplot displaying samples of essential oils using conductance change (Delta-G), slope, and area.

Figure 8 shows a biplot for the PCA analysis for the first two principal components. In the biplot, scores for the two essential oils correspond to the black symbols, and the loadings are represented by the red and blue symbols. The plot suggests that the information given by the slope (+) and the normalized conductance change (*) is somewhat anti-correlated, as their associated loadings appear at an angle of about 180 degrees across the center. In contrast, the information given by the area under the response curve (o) is somewhat orthogonal to both the information given by the slope and conductance change, as their loadings appear at an angle of about 90 degrees across the center. The loadings varied significantly from sensor to sensor, so it can be concluded that the six sensors used are useful for achieving a good discrimination performance.

#### 3.4.4. Discriminant Function Analysis

**Figure 9:**
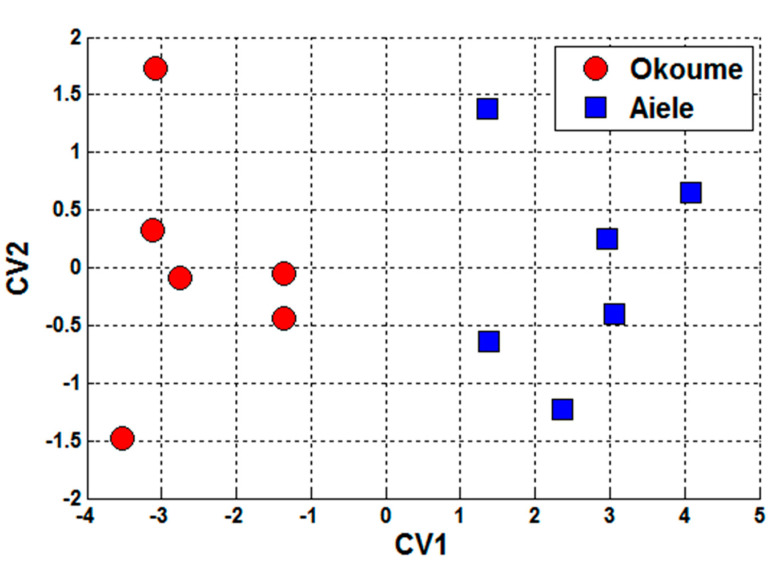
Discriminant function analysis (DFA) plot performed on essential oils, namely, okoume and aiele, using the conductance change (Delta-G) of sensors as a response feature.

The performance of the e-nose system in the classification of essential oil samples into two groups was evaluated applying DFA method. These groups correspond to those also separated by PCA. Figure 9 shows the DFA performed on two types of essential oils, okoume and aiele. We can see from this figure that all samples are perfectly separated from each other. These results confirm that the e-nose system was able to discriminate different types of essential oils that come from the same family plants.

#### 3.4.5. Hierarchical Cluster Analysis

**Figure 10:**
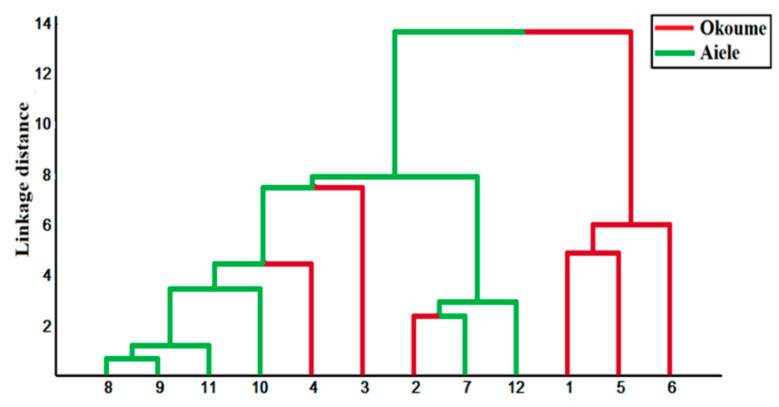
Hierarchical cluster analysis (HCA) dendrogram of the two essential oil samples measured using the conductance change (Delta-G) of sensors as a response feature.

The HCA provides an alternative for the visual representation of high-dimensional data. The results are illustrated in Figure 10 in the form of a dendrogram obtained from the essential oil samples by applying the Euclidean distance and Ward’s link to define the groups. At link. dist = 9, it can be seen that all the samples are grouped into two separate groups, corresponding to the essential oils analyzed. However, samples 2, 3, and 4 belonging to okoume oil are misclassified as aiele oil. The overlaps obtained may be due to the fact that the essential oils possess certain compounds in common according to the results obtained by GC-MS. This proves that the grouping classifications were highly correlated to the GC-MS results. In addition, this result does not contradict the PCA results in which okoume samples show a rather high dispersion in the score plot. Some okoume samples have scores that appear closer to the centroid for aiele, which possibly explains their misclassification by HCA.

## 4. Conclusions

The multi-strategy approach used in this study for the tasks of classification and detection of volatile bioactive compounds with antibacterial activity made it possible to highlight the differentiation of the samples of the essential oils studied. Moreover, a GC-MS technique was used to identify and qualitatively assess the content of bioactive compounds in the essential oil samples. As a result, twenty-one compounds for okoume and eighteen for aiele essential oil were identified. The main compounds were o-cymene(1-methyl-2-propan-2-ylbenzene), (+)-4-Carene ((1R,4S,6S)-4,7,7-trimethylbicyclo[4.1.0]hept-2-ene), 2-(4-methylcyclohex-3-en-1-yl)propan-2-ol for both essential oils. Benzenemethanol, α, α, 4-trimethyl-(28.85%), (+)-3-Carene (3,7,7-trimethyl bicyclo[4.1.0]hept-3-ene) (17.93%), D-Limonene ((4R)-1-methyl-4-prop-1-en-2-ylcyclohexene) (19.36%) for the okoume essential oil. 1-methyl-4-propan-2-yl-cyclohexa-1,4-diene (22.31%), 2-methyl-5-propan-2-yl bicyclo[3.1.0]hexan-2-ol (24.7%), (4-methyl-1-propan-2-yl cyclohex-3-en-1-yl) acetate (26.64%) for the aiele essential oil. On the one hand, the electronic nose, combined with several chemometric techniques, has demonstrated a great ability at discriminating between the two types of essential oils from the same plant family. Likewise, the results of the DFA lead to a satisfactory classification for all analyzed samples. In general, the essential oils have some common compounds according to the results obtained by GC-MS, which may explain the three misclassified samples when HCA was used. This proves that the grouping classifications of the e-nose were highly correlated with the GC-MS results. On the other hand, the combination of the e-nose system and GC-MS allowed the identification of bioactive compounds in the essential oil samples that have strong antimicrobial activity against the two bacteria tested, i.e., *S. aureus* and *E. coli*. This is because both essential oils contain monoterpenes, which have a strong antibacterial activity. Therefore, the use of such tools can ensure the determination of bioactive compounds in essential oils, which can be used against antibiotic-resistant bacteria.

## Figures and Tables

**Table 1:** Types of sensors used in the electronic nose and their principles.

Types of Sensors	Principles of Operation
TGS 815	High sensitivity and high selectivity to hydrogen
TGS 821	Highly sensitive to organic solvent vapors and a wide variety of combustible gases such as carbon monoxide
TGS 822	Organic solvents
TGS 824	Ammonia
TGS 825	Hydrogen sulfide
TGS 842	Methane, hydrocarbons (domestic gas alarm)

**Table 2:** Chemical composition of the essential oils of okoume and aiele, as identified by GC analysis.

VOC #	Chemical Species Name (IUPAC Name)	Essential Oil Samples Composition (%)	
		Aiele (*Canarium schweinfurthii*)	Okoume (*Aucoumea klaineana*)
1	((5S)-2,6,6-trimethyl bicyclo[3.1.1]hept-2-ene	12.41	0
2	2-methyl-5-propan-2-yl cyclohexa-1,3-diene	17.33	0
3	1-methyl-4-propan-2-yl cyclohexa-1,4-diene	22.31	0
4	2-methyl-5-propan-2-yl bicyclo[3.1.0]hexan-2-ol	24.70	0
5	(1S,4R)-4-Isopropyl-1-methyl-2-cyclohexen-1-ol	25.90	0
6	(4-methyl-1-propan-2-yl cyclohex-3-en-1-yl) acetate	26.64	0
7	1-methyl-4-propan-2-ylcyclohex-2-en-1-ol	26.83	0
8	2-[(1S)-4-methyl cyclohex-3-en-1-yl]propan-2-ol	29.03	0
9	Propan-2-one	0	1.63
10	Toluene	0	4.57
11	2,6,6-trimethyl bicyclo[3.1.1]hept-2-ene	0	12.45
12	7,7-dimethy l-2-methylidene bicyclo[2.2.1]heptane	0	13.19
13	2,2-dimethy l-3-methylidene bicyclo[2.2.1]heptane	0	13.28
14	6,6-dimethy l-2-methylidenebicyclo[3.1.1]heptane	0	15.12
15	3,7,7-trimethyl bicyclo[4.1.0]hept-3-ene	0	17.93
16	1-methyl-4-(propan-2-yl)benzene	0	19.26
17	(4R)-1-methyl-4-prop-1-en-2-ylcyclohexene	0	19.36
18	1-methyl-4-prop-1-en-2-yl cyclohexa-1,3-diene	0	24.91
19	Bicyclo[2.2. 1]heptan-2-one, 1,7,7-trimethyl-, (1S)-Camphor.	0	26.92
20	4-methyl-1-(propan-2-yl)cyclohex-3-en-1-ol	0	28.46
21	Benzenemethanol, α, α,4-trimethyl-	0	28.85
22	2-(4-methylcyclohex-3-en-1-yl)propan-2-ol	28.49	29.04
23	(1S)-2-methyl-5-propan-2-ylbicyclo[3.1.0]hex-2-ene	12.13	24.18
24	2,6,6-trimethyl bicyclo[3.1.1]hept-2-ene	12.45	12.09
25	1-methyl-2-propan-2-ylbenzene	14.80	18.83
26	4-methyl-1-propan-2-ylbicyclo[3.1.0]hex-2-ene	15.06	19.25
27	(1R,4S,6S)-4,7,7-trimethylbicyclo[4.1.0]hept-2-ene	24.07	16.88
28	1-methyl-4-propan-2-ylidenecyclohexane	24.18	22.77
